# Comparative Analysis of Duckweed Cultivation with Sewage Water and SH Media for Production of Fuel Ethanol

**DOI:** 10.1371/journal.pone.0115023

**Published:** 2014-12-17

**Authors:** Changjiang Yu, Changjiang Sun, Li Yu, Ming Zhu, Hua Xu, Jinshan Zhao, Yubin Ma, Gongke Zhou

**Affiliations:** 1 Key Laboratory of Biofuels, Qingdao Institute of Bioenergy and Bioprocess Technology, Chinese Academy of Sciences, Qingdao, 266101, PR China; 2 Beijing Risun Chemical Industry Technology Research institute Co. Ltd, Beijing, 100070, China; 3 Qingdao Institute of Animal Sciences, Qingdao, 266100, China; CEA-Saclay, France

## Abstract

Energy crises and environmental pollution have caused considerable concerns; duckweed is considered to be a promising new energy plant that may relieve such problems. *Lemna aequinoctialis* strain 6000, which has a fast growth rate and the ability to accumulate high levels of starch was grown in both Schenk & Hildebrandt medium (SH) and in sewage water (SW). The maximum growth rates reached 10.0 g DW m^−2^ day^−1^ and 4.3 g DW m^−2^ day^−1^, respectively, for the SH and SW cultures, while the starch content reached 39% (w/w) and 34% (w/w). The nitrogen and phosphorus removal rate reached 80% (SH) and 90% (SW) during cultivation, and heavy metal ions assimilation was observed. About 95% (w/w) of glucose was released from duckweed biomass hydrolysates, and then fermented by Angel yeast with ethanol yield of 0.19 g g^−1^ (SH) and 0.17 g g^−1^ (SW). The amylose/amylopectin ratios of the cultures changed as starch content increased, from 0.252 to 0.155 (SH) and from 0.252 to 0.174 (SW). *Lemna aequinoctialis* strain 6000 could be considered as valuable feedstock for bioethanol production and water resources purification.

## Introduction

Issues such as the rapid growth in demand for energy, rising energy prices, and environmental pollution concerns, and climate change, which is primarily caused by the large-scale use of fossil fuel energy, have led to mounting concerns about energy security throughout the world [1]. Searching for alternative sources of energy is becoming more and more important globally [2], [3]. The development of biofuels is one of the most effective ways to address and potentially ameliorate some of the problems with energy, the environment, and urbanization that governments are facing [2]. A new generation of biofuels, particularly bioethanol and microalgae biodiesel, which are produced from non-food biomass resources, have become the main direction of research into bio-liquid energy [4], [5].

Duckweed is one of the smallest but fastest-growing aquatic plants. The growth cycle of duckweed is very short (about 2∼3 days reproducing per generation). Duckweed grows at about 10 fold speed of that maize grows. Duckweed is commonly used for bioremediation of nutrient-rich wastewater and as fodder for fish and ducks due to its great ability in absorbing nutrients and high protein content [6]. Researches have recently demonstrated that duckweed can also be regarded as a potential feedstock for bioethanol production, because of its high levels of starch accumulation [7], [8]. The starch content of duckweed has been reported to be from 5%–70% of dry weight; starch accumulation in duckweed is known to be affected by many factors such as temperature, nutrient deprivation (mainly nitrogen and phosphorus), and light duration, all of which are factors that can be manipulated to increase starch content in experimental or production systems [9].

The conversion efficiency of starch-rich sources into ethanol is an additional problem that is currently receiving the attention of scientists and engineers [10]. Starch is a polymer of glucose that is composed of various genetically determined ratios of amylose and amylopectin [11]. Generally, amylose (MW = 160,000 D) consists of 500–20,000 glucose units joined by α-1,4 glycosidic bonds; amylopectin (MW = 32,400,000 D) is a branched polymer of glucose, that has, in addition to α-1,4 glycosidic bonds, branches (side chains) connected by α-1,6 glycosidic bonds [12]. The most common and important step for the recovery of glucose from starch is enzymatic hydrolysis, and most researches have shown that amylose content has a significant effect on ethanol fermentation efficiency. Typically, conversion efficiency decreases as the amylose content increases [13]–[15].

Due to the low cellulose and lignin content of duckweed, the biomass-to-ethanol conversion process is relatively easier and more cost-effective in duckweed than it is for other dedicated energy plants [16]. To date, the effect of amylose and amylopectin content in duckweed has not been investigated in the context of the production of ethanol or other bio-products. Additionally, there have been few studies that investigated the optimal growth conditions or water purification capabilities of duckweed cultivated with sewage water. In this study, the high-starch and rapid-growth *Lemna aequinoctialis* strain 6000 was cultured in Schenk & Hildebrandt medium (SH) and sewage water (SW) and evaluated for its utility in sewage water utilization and for its starch to ethanol conversion efficiency as a biofuel feedstock. Further, we measured the amylose and amylopectin content of duckweed and evaluated the impacts of these compounds on conversion efficiency. This study provides useful, foundational information that will help expand the application range of duckweed; such information will also be helpful in efforts to comprehensively understand the mechanisms that enable the relatively easy conversion of starch to ethanol that has been observed with duckweed as a feedstock.

## Materials and Methods

### Duckweed strains and culture conditions

*L. aequinoctialis* strain 6000, which has high starch content and rapid growth ability, was obtained through large scale screening of more than 100 strains of duckweeds distributed in 20 provinces and municipalities in China. These provinces and municipalities included Shanxi, Shandong, Guangxi, Guangdong, Henan, Hebei, Jiangxi, Jiangsu, Fujian, Zhejiang, Hainan, Shanxi, Hubei, Hunan, Sichuan, Guizhou, Beijing, Shanghai, Tianjin, and Chongqing. *L. aequinoctialis* strain 6000 was collected from Lixian in Hunan province (S1 Figure). No specific permits or legal permission were required for the collection of the duckweed species; the field studies did not involve endangered or protected species. The sewage water was provided by the Licun River sewage treatment factory in Qingdao. About 30 g of fresh duckweed plants, enough to cover the entire surface of the water with approximately a single layer of fronds, were put into a rectangular tank that was 60 cm long, 40 cm wide and 10 cm high. The duckweed plants were cultured in a growth chamber at 23°C under 16-h-light/8-h-dark conditions with 110 µmol m^−2^ s^−1^ irradiance. The diluted sewage water proportion was 1∶1 (wastewater: water). The liquid Schenk & Hildebrandt medium was supplemented with 10 g l^−1^ sucrose. Each treatment was cultured with three tanks.

### Growth rate and starch content measurement

The duckweed plants were harvested every 6 days for growth kinetics experiments. We drained the surface water with absorbent paper prior to measurement. The dry weight of duckweed was obtained by vacuum freeze drying for 48 h and then weighed on a precision balance. Then, total starch content of the dried plants was determined using the Megazyme total starch assay kit (Megazyme International, Ireland).

### Amylose/Amylopectin content assay

The amylopectin was precipitated for the amylose determination using Amylose/Amylopectin Assay Kits (Megazyme International, Ireland), according to the manufacturer's protocol. Amylopectin was determined by subtracting the amylose from the total starch. The detailed procedure is as follows: lyophilized duckweeds were milled in liquid nitrogen, and 50 mg of material was mixed with 2 ml of DMSO in a tube. These samples were heated in a boiling water bath for 15 min with intermittent stirring using a vortex mixer. 2 ml of DMSO was added to the mixture and 4 ml of Con A solvent was mixed in after the tube was bathed in boiling water for an additional 15 min. 1.0 ml of this solution was transferred to a tube to which 0.5 ml of Con A was added. The tube was allowed to stand for 1 hour at room temperature. The samples were then centrifuged at 12,000 rpm for 10 min at 20°C. 3 ml of 100 mM sodium acetate buffer (pH 4.5) was added to 1 ml of supernatant. The samples were mixed and heated in a boiling water bath for 5 min to denature the Con A, followed by a 5 min water bath at 40°C. 0.1 ml of amyloglucosidase/a-amylase enzyme mixture was mixed with the samples and incubated at 40°C for 30 min. The tube was then centrifuged at 3500 rpm for 6 min, of which 0.1 ml was added to 2 ml of glucose oxidase/peroxidase (GOPOD) reagent, vortexed, and incubated at 40°C for 20 min. Blank (1 ml of sodium acetate and 2 ml of GOPOD) and glucose standards were incubated concurrently at the following concentrations: 0.0, 0.02, 0.04, 0.06, 0.08 and 0.10 mg ml^−1^. The absorbance was measured with a spectrophotometer at 510 nm and 1 cm path length.

### Culture medium composition analysis

The nutrient level in the culture medium was determined by analyzing ammonia nitrogen (NH_4_-N) and total phosphorus (TP). Standard methods were used for the NH_4_-N (GB 7479-87) and the TP (GB 11893-89) analysis. Each treatment measurement was repeated three times. About 10 ml of SH and SW medium, from both before and after cultivation, were sampled via membrane filtration and the ion content in the medium was determined via inductively coupled plasma mass spectrometery (ICP-MS, PerkinElmer, USA) [17]. Dried *L. aequinoctialis* strain 6000 was ground in a pestle and 0.1 g of the powder was added to 5 ml of HNO_3_ and left to stand for 30 min. The samples were then placed in a Microwave Digestion System (CEM, USA) and digested for 25 min at 180°C and constant volume to 25 ml. Ion contents were determined by inductively coupled plasma mass spectrometery (ICP-MS, PerkinElmer, USA). Each treatment measurement was repeated three times.

### Enzymatic saccharification and sugar compositional analysis

A one-step hydrolysis process was used for enzymatic saccharification [16]. Briefly, 20 g of lyophilized duckweed was mixed with 80 ml of 25 mM NaOAc (pH = 5.5). The mixture was incubated at 100°C for 10 min, cooled down on ice, supplemented with 200 µl α-amylase (Sigma A4582), 150 µl α-amyloglucosidase (Sigma A7095), 150 µl pullulanase (Sigma P1067), and then incubated at 50°C with shaking at 250 rpm for 30 h.

Sugar compositional analysis was performed by high performance liquid chromatography (HPLC). Briefly, the hydrolysis products were derivatized with 1-phenyl-3-methyl-5-pyrazolone (PMP) and 0.3 M NaOH at 70°C for 30 min, extracted with chloroform three times, and then analyzed on a Hypersil ODS-2 C18 column (4.6×250 mm; Thermo Scientific) on a Waters HPLC System. PMP derivative (10 µl) was injected, eluted with 82% (v/v) phosphate buffer (0.1 M, pH 7.0) and 18% (v/v) acetonitrile at 1 ml min^−1^, and monitored with UV 245 nm. The monosaccharide standards used included fucose, rhamnose, arabinose, galactose, glucose, xylose, mannose, galacturonic acid, and glucuronic acid.

### Fermentation processes

Ethanol fermentations were carried out in bioreactors using Angel Yeast (Angel Yeast Co., Ltd, China), a yeast strain that is common and easily obtainable. Yeast cells were inoculated into 10 ml of each 100 ml hydrolysates (from the one-step hydrolysis) in the 250 ml flask. The bioreactors were placed in a shaking incubator and fermented at 30°C with shaking at 300 rpm for 30 h. The ethanol in the fermentation solution was measured with HPLC (Agilent 1200, USA).

### Statistical analysis

Data were presented as the mean ± standard deviation of the mean of triplicate samples. Significant differences between means were tested using one-way analysis of variance followed by least significant difference tests, using the SPSS statistical package (version 13.0; SPSS Inc., Chicago, IL, USA) at a significance level of *p*<0.05.

## Results and Discussion

### Duckweed growth and biomass production

**Figure 1:**
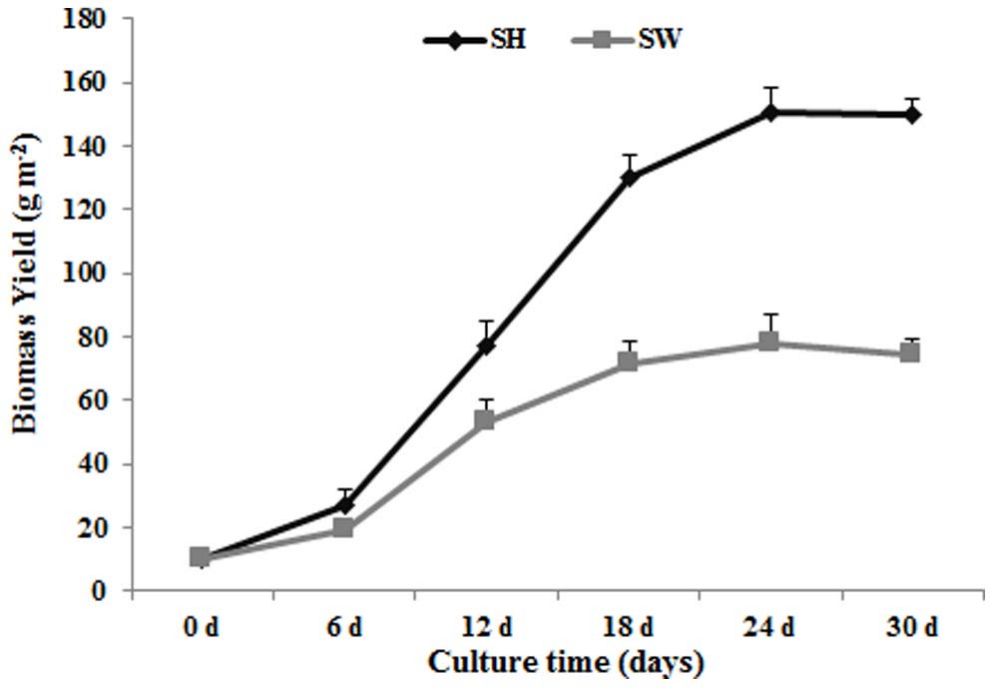
Kinetics of duckweed growth in Schenk & Hildebrandt medium (SH) and sewage water (SW). Each data point represents the mean of triplicate values; error bars indicate the standard deviation.

*L. aequinoctialis* was grown in diluted SW water and in SH medium for four weeks; SW provides suitable concentrations of nutrients, while SH provides ideal concentrations of nutrients. The biomass of duckweed plants grown in SW water increased by nearly 7.5 fold from an initial 10 g m^−2^ to a maximum of 77 g m^−2^ during the 18 days of cultivation, with a maximum growth rate of about 4.3 g DW m^−2^ day^−1^ (DW: dry weight) (Fig. 1). Following 18 days of the cultivation, the biomass did not increase further, indicating that the growth cycle for this strain of duckweed in SW culture was about 18 days. The biomass of the duckweed grown in SH medium increased about 15 fold over a period of 24 days, during which biomass increased from 10 g m^−2^ to about 150 g m^−2^, with a maximum growth rate of about 10 g DW m^−2^ day^−1^ (Fig. 1). Duckweed often demonstrates near exponential growth rates and many species have doubling times of 2 to 3 days, depending on the environmental conditions [6]. SH medium is an optimized culture medium for duckweed. The duckweed plants grown in the SH medium had a longer growth cycle due to proper nutrient ingredient. When grown in SW, the duckweed biomass was usually lower compared to that in the SH medium, due to the low nutrient levels and lack of sucrose. A previous report showed that the average growth rate of *Lemna minor* was 3.5 g DW m^−2^ day^−1^ when grown in swine lagoon wastewater, or 14.1 g DW m^−2^ day^−1^ when grown in SH medium [16]. It is thus important to select a duckweed strain and a proper cultivation time so that biomass production was closer between SH and SW. In large-scale cultivation, both cost and production capacity are important. SH medium is not economic for large-scale duckweed cultivation due to its high cost. SW, on the other hand, is nearly cost-free and its use can also bring enormous ecological/environmental benefits. Additionally, SH medium is not easy to prepare and extensive use of SH medium may lead to secondary water pollution because of its high inorganic element and sucrose content. The results of this study showed that duckweed biomass was lower in SW than in SH, which is consistent with previous reports. Nevertheless, *L. Aequinoctialis* still showed strong potential for application in biomass production using sewage water due to its lower cost for biomass production and enormous ecological/environmental benefits. According to our results, an annual output of the duckweed will be reached to 36.5 t DW ha^−1^ (SH) and 15.7 t DW ha^−1^ (SW). This is just in the lab condition where light density and nutrient was not enough. In the wild condition, we can use different resource wastewater mixed together which may supple enough nutrients and light intensity is higher, so we surmised that the annual output of the duckweed will be higher than the biomass of SH (36.5 t DW ha^−1^) cultivated by SW.

### Nutrient strength

**Figure 2:**
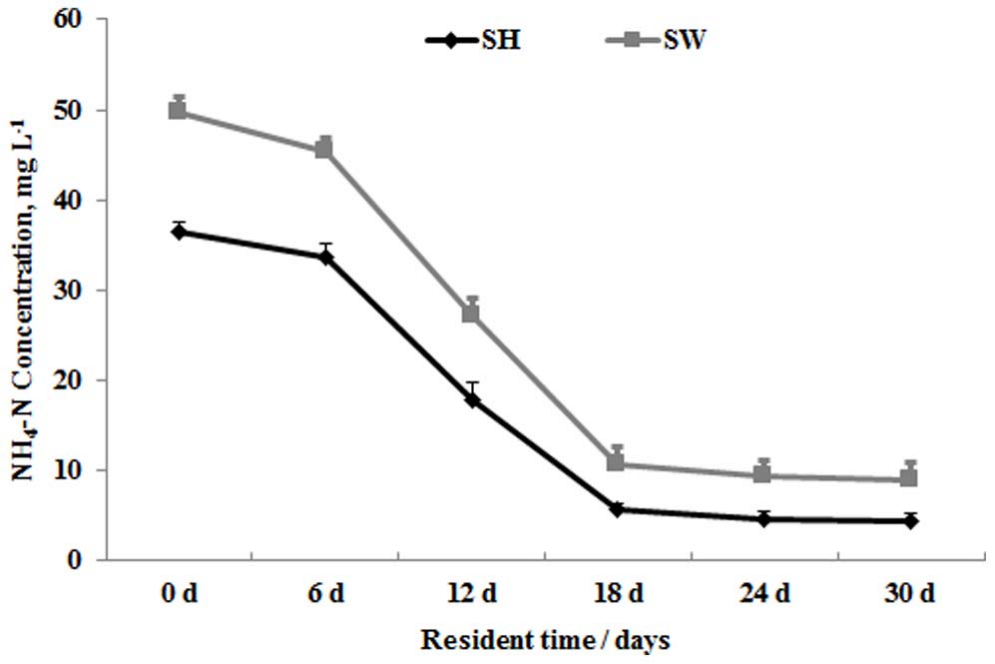
Duckweed-mediated nitrogen removal from Schenk & Hildebrandt medium (SH) and sewage water (SW). Each data point represents the mean of triplicate values; error bars indicate the standard deviation.

Nitrogen, phosphorus, and metal ions are the main nutrients which have an effect on water pollution, so the content of these was determined to evaluate the wastewater treatment capacity of *L. aequinoctialis*. NH_4_-N is the major organic nitrogen form after anaerobic treatment, so the NH_4_-N concentration during cultivation was measured to study the ability of nitrogen removal of *L. aequinoctialis*. Other inorganic nitrogen forms in the wastewater, including NO_3_-N and NO_2_-N were not measured due to their low content. As shown in Fig. 2, the concentration of NH_4_-N in SW of 50 mg l^−1^ was slightly higher than the 36 mg l^−1^ concentration of SH reaching, but the trend of the curves of nitrogen removal were similar in both treatments. The NH_4_-N concentration in SW and SH decreased slowly during the first 6 days and then fell off quickly over the next two weeks, with total nitrogen removal rate about 80%. The NH_4_-N concentration reached a low after cultivation for 18 days and changed little till the end of the period of cultivation.

**Figure 3:**
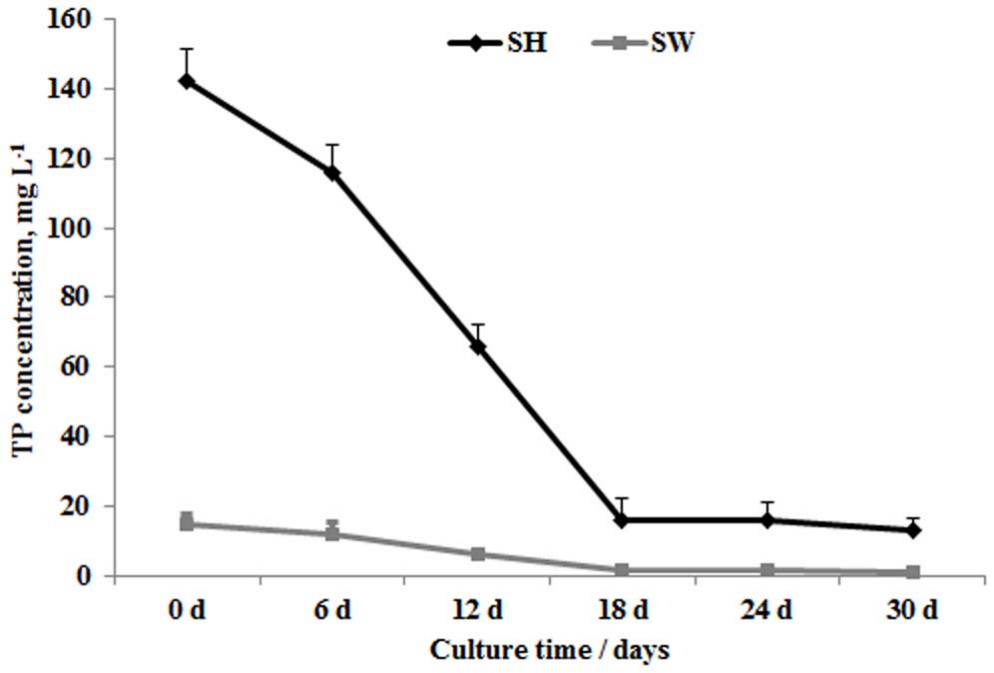
Changes in total phosphorus concentrations during duckweed cultivation in Schenk & Hildebrandt medium (SH) and sewage water (SW). Each data point represents the mean of triplicate values; error bars indicate the standard deviation.

Phosphorus is another major nutrient in wastewater; so total phosphorus (TP) was also measured during the culture period. As indicated in Fig. 3, *L. aequinoctialis* showed a highly efficient removal of phosphorus. The TP concentration in SH went from an initial 140 mg l^−1^ to 18 mg l^−1^ over 18 days. TP removal in the SW cultures showed the same trend, with the TP concentration dropping from 20 mg l^−1^ dropping to 1 mg l^−1^. The highest removal efficiency of TP for SH and SW were about 87% and 95%, respectively, by day 18. The TP concentrations in SH and SW changed little after 18 days of cultivation (Fig. 3).

Ion content in SH and SW were also determined before and after cultivation. As shown in Table 1, *L. aequinoctialis* absorbed a wide range of macro (P, K, Ca) to micro (Fe, Mn, Cu, Zn) elements. The concentration of phosphorus ions decreased by about 80% in SH over the course of cultivation and the phosphorus ions were almost completely absorbed in the SW treatment. These results were in accord with the TP result (Fig. 3) and thus provide further evidence that *L. aequinoctialis* has a high capacity for phosphorus removal. Sewage water usually contains heavy metals pollution. We therefore evaluated the Pb and Cd ion concentrations in the SW treatment. The results showed that before cultivation, the ion concentrations of Pb and Cd in buffered SW were 1.151 mg l^−1^ and 0.419 mg l^−1^, respectively (Table 1). After 30 days of cultivation, the Pb ion and Cd ion were both entirely removed and the heavy metal ions mainly transferred to the duckweed (Table 1), indicating that the selected *L. aequinoctialis* could not only efficiently remove NH_4_-N and TP, but also had a strong ability to remove heavy metal ions.

**Table 1:** Ion concentrations in Schenk & Hildebrandt medium (SH) and sewage water (SW) before and after cultivation.

	Ionic concentration (mg l^−1^)					
	before cultivation	after cultivation	before cultivation	after cultivation	before cultivation	after cultivation
Ion(mg l^−1^)	SH	SH	Buffered SW	Buffered SW	*Lemna* in SW	*Lemna* in SW
P	300.54±5.04	56.33±3.53	12.63±0.29	0.01±0.001	16.27±1.01	27.93±1.98
Mn	9.98±0.05	0.33±0.002	6.65±0.04	0.22±0.03	0.015±0.002	3.551±0.022
Zn	1.035±1.04	0.13±0.01	0.295±0.033	0.037±0.002	0.181±0.002	0.232±0.003
Cu	0.213±0.002	0.008±0.001	0.319±0.002	0.009±0.001	0.043±0.002	0.123±0.001
Fe	15.02±0.07	10.31±0.016	4.31±0.54	1.41±0.015	1.64±0.015	0.293±0.004
K	2505±12.94	1276.7±17.5	668.6±6.2	314±10.85	38.57±1.05	21.64±1.36
Ca	200.12±5.86	31.90±2.27	289.4±4.8	43.73±2.73	50.5±2.84	92.3±5.98
Na	45.51±6.57	110.56±15.62	223.2±5.3	166.33±4.67	4.853±0.874	26.78±1.95
B	4.986±0.05	0.032±0.007	1.653±0.011	0.028	1.705±0.361	0.729±0.036
Pb	0	0	1.151±0.009	0	0	1.101±0.011
Cd	0	0	0.419±0.001	0	0	0.413±0.002

There have been many studies using duckweed for nutrient recovery from swine wastewater [18]–[25]. For example, the duckweed system *Spirodela oligorrhiza* was capable of removing 83.7% and 89.4% of total nitrogen (TN) and TP respectively, from 6% swine lagoon water in eight weeks at a harvest frequency of twice a week [26]. *L. minor* was grown in agricultural wastewater for 20 days; the NH_4_-N removal rate reached 100% and the PO_4_-P removal rate reached 74.8% [16]. Beyond *S. oligorrhiza* and *L. minor*, the nutrient recovery abilities of *Wolffia arrhiza* and *Spirodela punctata* were also evaluated for agricultural wastewater [19], [24]. Although there have been many reports on the nutrient recovery ability of various duckweed species, most of the studies used agriculture wastewater such as swine lagoon water as the culture medium. In contrast, there have been few reports detailing the use of sewage water as the culture medium. Urban wastewater treatment is becoming more and more critical an environmental issue as urbanization rates increase around the world. Heavy metal pollution is a major problem in sewage water; it is difficult and expensive to remove heavy metal ions. Discharge of heavy metal ions into water can lead to great harm to plants and animals. Many duckweed species including *Wolffia globosa, L. minor* and *Lemna gibba* have been studied and considered as excellent candidates for heavy metal phytoremediation [27]–[29]. There is a dearth of data for duckweed heavy metal phytoremediation and nutrient removal using sewage water as the culture medium. We here showed that *L. aequinoctialis* not only efficiently removes nitrogen and phosphorus, but also has a good ability to remove heavy metal ions from sewage water. All of these results imply that *L. aequinoctialis* could be a useful duckweed species for pollution treatment and cultivation in sewage water.

### Starch accumulation

**Figure 4:**
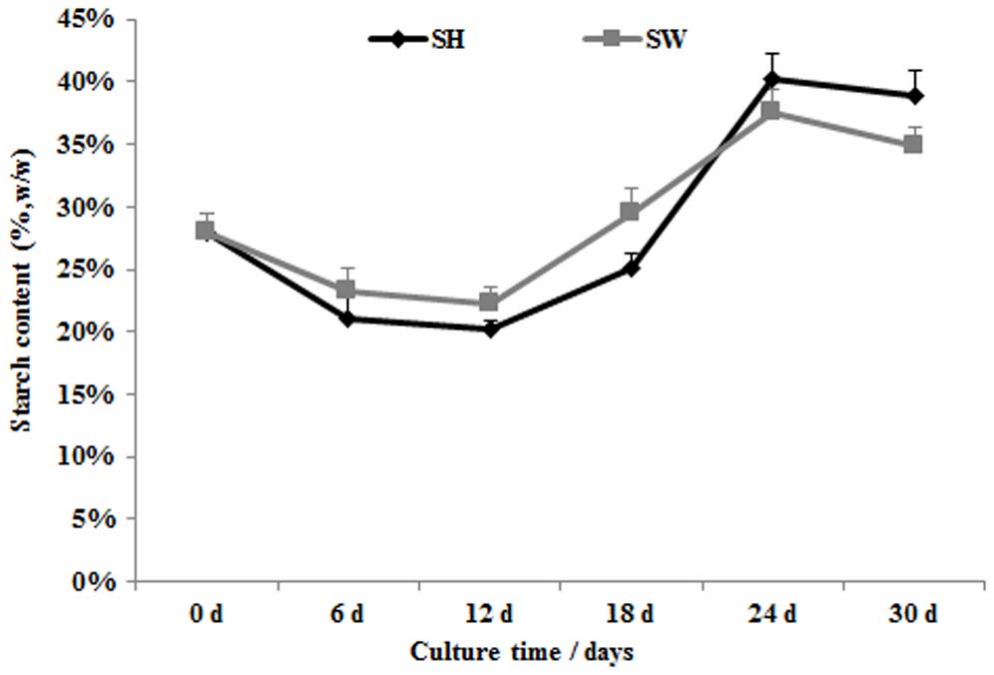
Kinetics of starch content variation during duckweed cultivation in Schenk & Hildebrandt medium (SH) and sewage water (SW). Each data point represents the mean of triplicate values; error bars indicate the standard deviation.

Starch accumulation tests were conducted every 6 days during the cultivation period. The initial starch content of *L. aequinoctialis* was 28% (w/w). This decreased slowly over the following 12 days. A common trend was observed: the starch content in plants in both the SH and SW treatments increased rapidly after 12 days and finally reached their highest levels of about 40% by 24 days (Fig. 4).

Many studies have shown that starch accumulation in duckweed can be induced by manipulating growth conditions such as nutrient levels, temperature, pH, light intensity and photoperiod [7]. In this study, the starch content decreased in the first 12 days, a result consistent with the biomass accumulation curve (Fig. 1). During the early growth stages of the cultivation period, duckweed biomass was rapidly increasing. But the starch content was not accumulation in the fast-growing phase. This phase can be seen in the biomass curve in the initial 12 days. An interesting observation was that the starch content of plants in SH was lower than duckweed in SW during day 12 to 18 (Fig. 4). Starch content of duckweed was affected by many factors, especially nutrient deficiency. Therefore, nutrient deficiency is usually way to induce starch accumulation. This work showed that duckweed in SH had higher biomass than SW in the fast-growing stage (on day 12), because there are more nutrients in SH than SW at this stage, duckweed may absorb more nutrients and consume more starch for the fast growth in SH. However, the SW nutrient is deficient at this stage, thus leading to accumulate starch content in SW. Therefore, starch content of SW was higher than SH in this stage. This should prove useful in large-scale duckweed production on SW. It showed that while biomass production in SW lower than production in SH, the most useful bioenergy content (starch) was higher in SW than in SH during day 12 to 18. Therefore, as starch production is not significantly different between the SH and SW culturing systems, growing *L. aequinoctialis* in SW is a highly relevant strategy for both bioremediation and the production of bioenergy feedstocks. The possibility is that the sufficient nutrients in the SH medium lead to higher biomass accumulation. After the fast-growing phase, the nutrient levels were decreased in both SH and SW, a condition that will induce starch accumulation. Nutrient starvation, especially N deficiency, is the most common strategy for enriching starch content in duckweed. Many studies have detailed the effect of nutrient deprivation on starch accumulation [26], [30]. The results that the starch content curve was consistent with early reports showed the starch induction of content after 18 days when the nutrient of the medium such as nitrogen and phosphors in SH and SW were almost totally adsorbed (Fig. 4). The starch percentage of duckweed ranges from 3% to 75% (DW), depending on the duckweed species and growing conditions [9]. The starch content of *L. aequinoctialis* was about 28%, though it reached more than 40% after nutrient deprivation (Fig. 4). In addition to its fast growth ability, *L. aequinoctialis* showed great potential as a feedstock for producing starch-based products such as fuel ethanol.

### Bioethanol production

The duckweed harvested from the SH and SW treatments had final starch content of 39% and 34%, respectively (Table 2). Enzymatic hydrolysis is the most common and important step for the recovery of glucose from starch. The common process used with starch to obtain glucose is a two-step process in which the cellulose and starch fractions are hydrolyzed at different pH and temperature conditions. However, according to early research [16], one-step hydrolysis releases even more glucose at similar solid-loading levels than does the two-step procedure. So one-step procedure was used from a low solid-loading for enzymatic hydrolysis. After the enzymatic hydrolysis of duckweed biomass at solid loading (20 g DW) using α-amylase, α-amyloglucosidase, and pullulanase, reducing sugar recovery reached 94.14% in SH medium (8.65 g l^-1^) and 94.63% in SW (7.58 g l^−1^) (Table 3). The reducing sugar components of hydrolysis products were measured with HPLC and the result showed that glucose was the major component of hydrolysis, accounting for 94% of reducing sugar in SH and 96% in SW. Other sugars such as galactose and mannose accounted for only 6% of reducing sugar in SH and only 4% in SW (Table 4).

**Table 2:** Changes in the amylose, amylopectin, and starch content of *L. aequinoctialis* before and after cultivation in Schenk & Hildebrandt medium (SH) and sewage water (SW).

Duckweed starch characteristics		After cultivation	
	Control	SH	SW
Amylose (%)	20.12±1.05^a^	13.40±1.48^b^	14.83±0.61^b^
			
Amylopectin (%)	79.88±1.05^b^	86.60±1.48^a^	85.17±0.61^a^
			
Amylose/Amylopectin	0.252±0.02^a^	0.155±0.01^b^	0.174±0.02^b^
			
Total starch (%)	28±1.44^c^	39±1.95^a^	34±1.62^b^
			

All data are presented as the mean of triplicate measurements ± standard deviation. Different letters indicate significant differences between different conditions (*p*<0.05).

**Table 3:** Glucose released from the biomass of duckweed after enzymatic saccharification and ethanol yields of the fermentation in hydrolysates of duckweed biomass with Angel Yeast.

Saccharification and Fermentation	SH	SW
		
Biomass input (g DW l^−1^)	19.98±0.4	19.99±0.3
Total starch content (g l^−1^)	9.19±0.2	8.01±0.2
Released glucose (g l^−1^)	8.65±0.1	7.58±0.2
Release percentage (%)	94.14±0.5	94.63±0.4
Y*_E_*(g)	3.80±0.2	3.38±0.3
Y*_E/G_* (g g^−1^)	0.44±0.04	0.45±0.02
Y*_E/B_* (g g^−1^)	0.19±0.04	0.17±0.03

Each data is the mean of three replicates ± standard deviation.

**Table 4:** Reducing sugar analysis of hydrolyzates of duckweed grown in Schenk & Hildebrandt medium (SH) and sewage water (SW). Glc: glucose, Gal: galactose, Man: mannose.

	SH	SW
Glc	94%±2%^a^	96%±6%^a^
Gal	2%±0.2%^b^	1%±0.3%^b^
Man	4%±0.4%^b^	3%±0.1%^b^

Different letters indicate significant differences between different conditions (*p*<0.05)

In the fermentation of the hydrolysates, a common yeast strain (Angel yeast) used to evaluate the fermentation ability of duckweed. In the final fermentation broth, 3.8 g and 3.38 g of ethanol were detected with the 8.65 g l^−1^ and 7.58 g l^−1^ initial glucose concentrations, respectively (Table 3). The ATCC 24859 strain has been used as model yeast for fermenting starch to ethanol in both maize and duckweed [7], [31]. Some yeast with special features for fermentation have also been studied for use with duckweeds. An example of this is the SPSC01 yeast strain that has a high tolerance to ethanol and fermentation inhibitors [32]. According to Ge et al., no significant difference (*p*<0.05) in ethanol yield (based on glucose, Y_E/G_) was found between using the ATCC 24859 strain and SPSC01 yeast strain [16]. Therefore, in order to reduce costs and simplify the procedure, we selected a common yeast strain that also had good fermentation characteristics. Compared to ATCC 24859, Angel yeast is cheaper and easier to obtain and thus more suitable for large scale ethanol production.

The ethanol yields Y_E/G_ = 0.44–0.45g g^−1^ obtained from this experiment showed that Angel yeast is also fit for fermenting starch to ethanol from duckweed (Table 3). The ethanol yields Y_E/B_ = 0.17–0.19 g g^−1^ in our duckweed biomass fermentation experiments were higher than those reported earlier for the conversion of various terrestrial plant biomass feedstocks to ethanol (0.122–0.180 g g^−1^) (Table 3) [32], [33]. An additional advantage is that, *L. aequinoctialis* does not require harsh pretreatment (s) or vigorous extraction/washing to remove inhibitors. These also indicated that duckweed had good potential as a bioenergy resource. It has been reported that duckweed has the potential to accumulate starch up to 60–70% DW (w/w), so it is conceivable that the ethanol yields from duckweed feedstock could reach even higher levels after optimized cultivation parameters for starch content accumulation.

### Amylose/amylopectin

**Figure 5:**
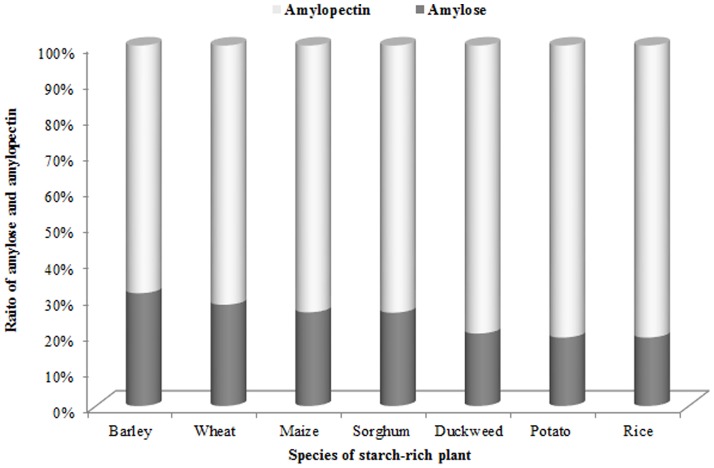
The ratio of amylose to amylopectin in duckweed and other starch-rich crops [34].

In order to learn more about the favorable fermentation characteristics of duckweed, we measured the amylose and amylopectin content before and after cultivation. As shown in Table 2, prior to cultivation, the content of amylose in *L. aequinoctialis* was 20.12% and the amylopectin content was nearly 80%, with a ratio of 0.252. The amylose content of duckweed is lower than that of other starch-rich crops such as barley, wheat, maize, and sorghum; the amylose content of duckweed is similar to that of potato and rice (Fig. 5) [34]. Further, our result showed that the amylose/amylopectin ratio changed during cultivation; a finding that has not been reported for other plants. That is, *L. aequinoctialis* preferentially biosynthesized amylopectin following the induction of starch accumulation. After cultivation, the amylose/amylopectin ratios in SH and SW were 0.155 and 0.174, respectively (Table 2).

It is known that amylose content has a significant effect on ethanol fermentation efficiency; conversion efficiency decreases as amylose content increases [13]. For example, the digestibility of rice starch was negatively correlated with the amylose content in rice starch [35]. Other studies have obtained similar results when examining the amylolytic hydrolysis of waxy, normal, and high-amylose starches of maize [11]. It has been customary to say that duckweed is amenable to hydrolysis and fermentation due to its low cellulose content and lack of lignin, but very little research about amylose and amylopectin content in duckweed has been reported. We speculate that the composition of starch in duckweed is also an important characteristic of the relatively easy hydrolysis and fermentation of duckweed feedstocks. In our study, *L. aequinoctialis* had lower amylose content (20.12%) and higher amylopectin content (79.88%). In the end of the cultivation, the amylopectin content reached 86.6% (SH) and 85.17% (SW) (Table 2). It has been suggested that the increased starch content correlates with increased amylopectin content, a situation that would be advantageous for bioethanol production. To the best of our knowledge, this study is the first to measure the amylose and amylopectin content in duckweed.

## Conclusions

A high starch content duckweed *L. aequinoctialis* strain 6000 revealed a great potential for bioremediation of sewage water. This species had good nitrogen and phosphorus removal ability and also fast rates of growth in either SH or SW media. *L. aequinoctialis* also had good heavy metal ion adsorption capacity which suggests that it is an excellent choice for use in sewage water treatment applications. The phenomenon that higher amylopectin content has an effect on fermentation was first found in duckweed. These findings provide a basis for novel speculation about the reason that duckweed can easily undergo amylolytic hydrolysis in addition to the obvious reasons such as lack of lignin and deficiency in hemicellulose. And the amylose/amylopectin ratio also provides further evidence that duckweed is an ideal plant as a feedstock for easy and cost-effective biomass-to-ethanol conversion. After enzymatic saccharification and fermentation, the starch in *L. aequinoctialis* was easily and efficiently converted to ethanol. The ultimate goal is that this research will contribute to the development of the use of duckweed for nutrient recovery and bioethanol production in sewage water system management.

## Supporting Information

S1 Figure **Collection of duckweed species distributed across 20 provinces and municipalities in China.** The provinces and municipalities from which samples were collected are marked with black stars.
(EPS)

## References

[1] Qiu H, Huang J, Keyzer M, van Veen W, Rozelle S, et al (2011) Biofuel development, food security and the use of marginal land in China. J Environ Qual 40:1058–1067. 21712574 10.2134/jeq2011.0012

[2] Ragauskas AJ, Williams CK, Davison BH, Britovsek G, Cairney J, et al (2006) The path forward for biofuels and biomaterials. Science 311:484–489. 16439654 10.1126/science.1114736

[3] Searchinger T, Heimlich R, Houghton RA, Dong F, Elobeid A, et al (2008) Use of U.S. croplands for biofuels increases greenhouse gases through emissions from land-use change. Science 319:1238–1240. 18258860 10.1126/science.1151861

[4] Wyman CE (2007) What is (and is not) vital to advancing cellulosic ethanol. Trends Biotechnol 25:153–157. 17320227 10.1016/j.tibtech.2007.02.009

[5] Scott SA, Davey MP, Dennis JS, Horst I, Howe CJ, et al (2010) Biodiesel from algae: challenges and prospects. Curr Opin Biotechnol 21:277–286. 20399634 10.1016/j.copbio.2010.03.005

[6] Stomp AM (2005) The duckweeds: a valuable plant for biomanufacturing. Biotechnol Annu Rev 11:69–99. 16216774 10.1016/S1387-2656(05)11002-3

[7] Xu JL, Cui WH, Cheng JJ, Stomp AM (2011) Production of high-starch duckweed and its conversion to bioethanol. Biosyst Eng 110:67–72.

[8] Xiao Y, Fang Y, Jin YL, Zhang GH, Zhao H (2013) Culturing duckweed in the field for starch accumulation. Ind Crop Prod 48:183–190.

[9] Cheng JJ, Stomp AM (2009) Growing Duckweed to Recover Nutrients from Wastewaters and for Production of Fuel Ethanol and Animal Feed. Clean-Soil Air Water 37:17–26.

[10] Lin Y, Tanaka S (2006) Ethanol fermentation from biomass resources: current state and prospects. Appl Microbiol Biotechnol 69:627–642. 16331454 10.1007/s00253-005-0229-x

[11] Tester RF, Karkalas J, Qi X (2004) Starch-composition, fine structure and architecture. J Cereal Sci 39:151–165.

[12] Murthy GS, Johnston DB, Rausch KD, Tumbleson ME, Singh V (2011) Starch hydrolysis modeling: application to fuel ethanol production. Bioprocess Biosyst Eng 34:879–890. 21487699 10.1007/s00449-011-0539-6

[13] Wu X, Zhao R, Wang D, Bean SR, Seib PA, et al (2006) Effects of amylose, corn protein, and corn fiber contents on production of ethanol from starch-rich media. Cereal Chem 83:569–575.

[14] Ai Y, Medic J, Jiang H, Wang D, Jane JL (2011) Starch characterization and ethanol production of sorghum. J Agric Food Chem 59:7385–7392. 21604720 10.1021/jf2007584

[15] Yangcheng H, Jiang H, Blanco M, Jane JL (2013) Characterization of normal and waxy corn starch for bioethanol production. J Agric Food Chem 61:379–386. 23241103 10.1021/jf305100n

[16] Ge X, Zhang N, Phillips GC, Xu J (2012) Growing *Lemna minor* in agricultural wastewater and converting the duckweed biomass to ethanol. Bioresour Technol 124:485–488. 22985823 10.1016/j.biortech.2012.08.050

[17] O'Sullivan JE, Watson RJ, Butler EC (2013) An ICP-MS procedure to determine Cd, Co, Cu, Ni, Pb and Zn in oceanic waters using in-line flow-injection with solid-phase extraction for preconcentration. Talanta 115:999–1010. 24054694 10.1016/j.talanta.2013.06.054

[18] Bergmann BA, Cheng J, Classen J, Stomp AM (2000) In vitro selection of duckweed geographical isolates for potential use in swine lagoon effluent renovation. Bioresour Technol 73:13–20.

[19] Cheng JY, Bergmann BA, Classen JJ, Stomp AM, Howard JW (2002) Nutrient recovery from swine lagoon water by *Spirodela punctata* . Bioresour Technol 81:81–85. 11708759 10.1016/s0960-8524(01)00098-0

[20] Cheng J, Landesman L, Bergmann BA, Classen JJ, Howard JW, et al (2002) Nutrient removal from swine lagoon liquid by *Lemna minor* 8627. T Asae 45:1003–1010.

[21] Chaiprapat S, Cheng JJ, Classen JJ, Liehr SK (2005) Role of internal nutrient storage in duckweed growth for swine wastewater treatment. T Asae 48:2247–2258.

[22] El-Shafai SA, El-Gohary FA, Nasr FA, van der Steen NP, Gijzen HJ (2007) Nutrient recovery from domestic wastewater using a UASB-duckweed ponds system. Bioresour Technol 98:798–807. 16713255 10.1016/j.biortech.2006.03.011

[23] Xu J, Shen G (2011) Effects of harvest regime and water depth on nutrient recovery from swine wastewater by growing *Spirodela oligorrhiza* . Water Environ Res 83:2049–2056. 22195427 10.2175/106143011x12989211841377

[24] Suppadit T (2011) Nutrient removal of effluent from quail farm through cultivation of *Wolffia arrhiza* . Bioresour Technol 102:7388–7392. 21669519 10.1016/j.biortech.2011.05.061

[25] Mohedano RA, Costa RH, Tavares FA, Belli Filho P (2012) High nutrient removal rate from swine wastes and protein biomass production by full-scale duckweed ponds. Bioresour Technol 112:98–104. 22425517 10.1016/j.biortech.2012.02.083

[26] Xu J, Shen G (2011) Growing duckweed in swine wastewater for nutrient recovery and biomass production. Bioresour Technol 102:848–853. 20869239 10.1016/j.biortech.2010.09.003

[27] Khellaf N, Zerdaoui M (2010) Growth response of the duckweed *Lemna gibba L.* to copper and nickel phytoaccumulation. Ecotoxicology 19:1363–1368. 20680456 10.1007/s10646-010-0522-z

[28] Üçüncü E, Tunca E, Fikirdeşici Ş, Altındağ A (2013) Decrease and Increase Profile of Cu, Cr and Pb during Stable Phase of Removal by Duckweed (*Lemna minor L*.). Int J Phytoremediat 15:376–384. 10.1080/15226514.2012.702808 23488003

[29] Xie WY, Huang Q, Li G, Rensing C, Zhu YG (2013) Cadmium Accumulation in the Rootless Macrophyte *Wolffia globosa* and Its Potential for Phytoremediation. Int J Phytoremediat 15:385–397. 10.1080/15226514.2012.702809 23488004

[30] Xu JL, Cheng JJ, Stomp AM (2012) Growing *Spirodela polyrrhiza* in Swine Wastewater for the Production of Animal Feed and Fuel Ethanol: A Pilot Study. Clean-Soil Air Water 40:760–765.

[31] Shrestha P, Khanal SK, Pometto AL 3rd, Hans van Leeuwen J (2010) Ethanol production via in situ fungal saccharification and fermentation of mild alkali and steam pretreated corn fiber. Bioresour Technol 101:8698–8705. 20624677 10.1016/j.biortech.2010.06.089

[32] Ge X, Burner DM, Xu J, Phillips GC, Sivakumar G (2011) Bioethanol production from dedicated energy crops and residues in Arkansas, USA. Biotechnol J 6:66–73. 21086455 10.1002/biot.201000240

[33] Chen Y, Sharma-Shivappa RR, Keshwani D, Chen C (2007) Potential of agricultural residues and hay for bioethanol production. Appl Biochem Biotechnol 142:276–290. 18025588 10.1007/s12010-007-0026-3

[34] Wang K, Hasjim J, Wu AC, Henry RJ, Gilbert RG (2014) Variation in Amylose Fine Structure of Starches from Different Botanical Sources. J Agric Food Chem 62:4443–453. 24758598 10.1021/jf5011676

[35] Okuda M, Aramaki I, Koseki T, Satoh H, Hashizume K (2005) Structural characteristics, properties, and in vitro digestibility of rice. Cereal Chem 82:361–368.

